# Endocrine-Disrupting Chemicals and Oil and Natural Gas Operations: Potential Environmental Contamination and Recommendations to Assess Complex Environmental Mixtures

**DOI:** 10.1289/ehp.1409535

**Published:** 2015-08-27

**Authors:** Christopher D. Kassotis, Donald E. Tillitt, Chung-Ho Lin, Jane A. McElroy, Susan C. Nagel

**Affiliations:** 1 Nicholas School of the Environment, Duke University, Durham, North Carolina, USA; 2 U.S. Geological Survey, Columbia Environmental Research Center, Columbia, Missouri, USA; 3 Department of Forestry, School of Natural Resources, University of Missouri, Columbia, Missouri, USA; 4 Department of Family and Community Medicine, and; 5 Department of Obstetrics, Gynecology and Women’s Health, School of Medicine, University of Missouri, Columbia, Missouri, USA

## Abstract

**Background:**

Hydraulic fracturing technologies, developed over the last 65 years, have only recently been combined with horizontal drilling to unlock oil and gas reserves previously deemed inaccessible. Although these technologies have dramatically increased domestic oil and natural gas production, they have also raised concerns for the potential contamination of local water supplies with the approximately 1,000 chemicals that are used throughout the process, including many known or suspected endocrine-disrupting chemicals.

**Objectives:**

We discuss the need for an endocrine component to health assessments for drilling-dense regions in the context of hormonal and antihormonal activities for chemicals used.

**Methods:**

We discuss the literature on *a*) surface and groundwater contamination by oil and gas extraction operations, and *b*) potential human exposure, particularly in the context of the total hormonal and antihormonal activities present in surface and groundwater from natural and anthropogenic sources; we also discuss initial analytical results and critical knowledge gaps.

**Discussion:**

In light of the potential for environmental release of oil and gas chemicals that can disrupt hormone receptor systems, we recommend methods for assessing complex hormonally active environmental mixtures.

**Conclusions:**

We describe a need for an endocrine-centric component for overall health assessments and provide information supporting the idea that using such a component will help explain reported adverse health trends as well as help develop recommendations for environmental impact assessments and monitoring programs.

**Citation:**

Kassotis CD, Tillitt DE, Lin CH, McElroy JA, Nagel SC. 2016. Endocrine-disrupting chemicals and oil and natural gas operations: potential environmental contamination and recommendations to assess complex environmental mixtures. Environ Health Perspect 124:256–264; http://dx.doi.org/10.1289/ehp.1409535

## Introduction

A novel source of human and animal exposure to endocrine-disrupting chemicals (EDCs) is through their use in oil and gas drilling operations. EDCs are exogenous compounds that can disrupt both development and normal hormone action either directly, by interacting with hormone receptors as agonists/antagonists, or indirectly by, for example, altering endogenous hormone concentrations, delivery to receptors, modulation of endogenous hormone responses, enzyme activities, or other mechanisms (Bergman et al. 2013; Diamanti-Kandarakis et al. 2009; Zoeller et al. 2014). Importantly, oil and gas operation chemicals have been shown to act through both direct and indirect mechanisms (Andric et al. 2006; Kassotis et al. 2014; Knag et al. 2013; Thomas and Budiantara 1995). EDCs can exhibit effects at extremely low, environmentally relevant concentrations, particularly during sensitive windows when exposure can alter normal development and result in adverse health outcomes during adulthood (Vandenberg 2014; Vandenberg et al. 2012; vom Saal et al. 2007; Welshons et al. 2003). Although chemicals used in and produced by oil and gas operations include EDCs, carcinogens, radioactive compounds, and other toxicants, herein, we will focus on the unique issues posed by their endocrine-disrupting activities.

In hydraulic fracturing, millions of gallons of water, tens of thousands of gallons of chemicals, and millions of kilograms of suspended solids are injected into the ground under high pressure. Hydraulic fracturing serves to fracture the shale or coal bed layer and release trapped natural gas or oil, allowing for increased well production. Although hydraulic fracturing technologies have been developed over the last 65 years, they have only recently been combined with horizontal drilling to unlock vast new oil and gas reserves around the world that were previously deemed either inaccessible or unprofitable (Waxman et al. 2011; Wiseman 2008). Chemicals are added throughout the entire production process (including drilling, fracturing, and through closure) for a number of reasons (Table 1) (Deutch et al. 2011; Riedl et al. 2013; Waxman et al. 2011). In total, approximately 1,000 chemicals are known to be used throughout the process [U.S. Environmental Protection Agency (EPA) 2015; Waxman et al. 2011].

**Table 1:** Functional categories of hydraulic fracturing chemicals [adapted from Colborn et al. (2011)].

Chemical categories	Technical hydraulic fracturing use	Example compounds
Acids	To achieve greater injection ability or penetration and later to dissolve minerals and clays to reduce clogging, allowing gas to flow to the surface.	Hydrochloric acid
Biocides	To prevent bacteria that can erode pipes and fittings and to break down gellants that serve to ensure that fluid viscosity and proppant transport are maintained.	1-methyl-4-isothiazolin-3-one, bronopol, glutaraldehyde
Breakers	To allow the breakdown of gellants used to carry the proppant; these are added near the end of the hydraulic fracturing sequence to enhance flowback.	Ammonium persulfate, magnesium peroxide
Clay stabilizers	To create a fluid barrier to prevent mobilization of clays, which can plug fractures.	Tetramethyl ammonium chloride, sodium chloride
Corrosion inhibitors	To reduce the potential for rusting in pipes and casings.	Ethoxylated octylphenol and nonylphenol, isopropanol
Crosslinkers	To thicken fluids, often with metallic salts, in order to increase viscosity and proppant transport.	Ethylene glycol, sodium tetraborate decahydrate, petroleum distillate
Defoamers	To reduce foaming after it is no longer needed in order to lower surface tension and allow trapped gas to escape.	2-ethylhexanol, oleic acid, oxalic acid
Foamers	To increase carrying capacity while transporting proppants and decreasing the overall volume of fluid needed.	2-butoxyethanol, diethylene glycol
Friction reducers	To make water slick and minimize the friction created under high pressure and to increase the rate and efficiency of moving the hydraulic fracturing fluid.	Acrylamide, ethylene glycol, petroleum distillate, methanol
Gellants	To increase viscosity and suspend sand during proppant transport.	Propylene glycol, guar gum, ethylene glycol
pH control	To maintain the pH at various stages with buffers to ensure the maximum effectiveness of various additives.	Sodium hydroxide, acetic acid
Proppants	To hold fissures open, allowing gas to flow out of the cracked formation; usually composed of sand and occasionally glass or ceramic beads.	Styrene, crystalline silica, ceramic, graphite
Scale inhibitors	To prevent buildup of mineral scale that can block fluid and gas passage through the pipes.	Acrylamide, sodium polycarboxylate
Surfactants	To decrease liquid surface tension and improve fluid passage through pipes in either direction.	Naphthalene, 1,2,4-trimethylbenzene, ethanol, methanol, 2-butoxyethanol
Categories and uses for commonly applied chemicals that are commonly used throughout the hydraulic fracturing process with specific examples provided for each category class. Adapted with permission from Colborn T et al. (2011). Reprinted by permission of Taylor & Francis LLC (http://www.tandfonline.com).		

Following the initial injection into the well to generate fractures, a portion of the injected volume returns to the surface immediately; this fluid is known as “flow-back.” The remaining fluids either permeate the shale or coal bed formation and/or return to the surface over the life of the producing well; this fluid is known as “produced water.” Both types of wastewater can contain fracturing fluids, naturally occurring salts, radioactive materials, heavy metals, and other chemicals from the shale formation such as polycyclic aromatic hydrocarbons, alkenes, alkanes, and other volatile and semi-volatile organic compounds (Deutch et al. 2011; Fontenot et al. 2013; Harkness et al. 2015; Harvey et al. 1984; Maule et al. 2013; Warner et al. 2012). Wastewater is disposed of via injection wells, open evaporation pits, landfills, or treatment plants; through on-site burial; by being spread over road or fields; and/or by being treated and reused in future hydraulic fracturing operations (Deutch et al. 2011; Gilmore et al. 2014; Lee et al. 2011; Wiseman 2008). Treatment of wastewater for reuse or disposal varies by geological region owing to differing chemical compositions and may include biological treatment, filtration or aeration steps, and/or reverse-osmosis separation (Lester et al. 2015).

## Potential Routes of Exposure to Oil and Natural Gas Operation Chemicals

*Water.* Oil and natural gas operations can lead to the contamination of surface and groundwater, both of which are sources of drinking water (reviewed by Brantley et al. 2014; Burton et al. 2014; Vengosh et al. 2014). There are a variety of routes of contamination: spills of chemicals during transport to and from the fracturing site, the drilling and fracturing processes, improper treatment and disposal of wastewater, failure of well casings, and structural failure in abandoned wells (Ingraffea et al. 2014; Kell 2011; Mauter et al. 2014; Rozell and Reaven 2012).

In 2013, spills were reported at 1% of Colorado wells (550/51,000 active wells), and it has been estimated that 50% of surface spills contaminate groundwater on the basis of data from Weld County, Colorado (Gross et al. 2013). An analysis of permitted Pennsylvania wells suggests a similar total spill rate of 2% (103/5,580 active wells; Souther et al. 2014). Although all 24 states with active shale reservoirs report spills, reporting limits and required information vary widely, and only 5 states require maintenance of public records for spills and violations (Soraghan 2014; Souther et al. 2014). Given the limited mandatory reporting, it is likely that the magnitude of the impact of oil and gas operations on water quality is underestimated (Soraghan 2014; Souther et al. 2014). For example, an analysis in Pennsylvania found that industry had reported 59% of documented spills (Souther et al. 2014).

Wastewater is commonly sent to wastewater treatment plants in many regions (Gilmore et al. 2014) that are not able to remove many of the anthropogenic or naturally occurring compounds present in wastewater from shale operations (Braga et al. 2005; Campbell et al. 2006; Westerhoff et al. 2005). Following this treatment, these compounds can be discharged into surface water (Ferrar et al. 2013b; Harkness et al. 2015; Warner et al. 2013, 2014). Transportation of chemicals for drilling and fracturing to well pads and transportation of wastewater away from well pads poses risks for contamination (Burton et al. 2014). Spills and leaks occur during transportation through wastewater pipelines, transfer to trucks at well pads, and vehicular transport to disposal facilities (Gilmore et al. 2014).

Groundwater contamination associated with oil and gas operations has also been reported (Fontenot et al. 2013; Jackson et al. 2013; Osborn et al. 2011; Vengosh et al. 2014). This contamination can occur via migration of chemicals from the surface or underground. An investigation of wastewater pits and impoundments in the Marcellus Shale region reported a lack of maintenance of containment and transport systems, with spills affecting groundwater largely as a result of equipment failures and corrosion of pipes and tanks (Ziemkiewicz et al. 2014). Surface spills of fracturing fluids can also contaminate groundwater, and elevated concentrations of benzene, toluene, ethylbenzene, and xylenes (BTEX) have been reported in groundwater near surface spills (Gross et al. 2013; Ziemkiewicz et al. 2014). A recent U.S. EPA report conclusively linked hydraulic fracturing to drinking-water contamination at wells within five of six retrospective study regions; no baseline testing was available for the sixth region (U.S. EPA 2015). Underground migration potential is also a concern. Concentrations of heavy metals have been shown to increase in drinking water with proximity to natural gas wells (Fontenot et al. 2013), and thermogenic (shale-origin) gas concentrations in drinking water sampled from close proximity to natural gas wells have been reported to be higher than in water sampled from more distant sources (Jackson et al. 2013; Li and Carlson 2014; Osborn et al. 2011). Recent work suggests that the main reason for these findings may be faulty well casings (Darrah et al. 2014).

*Air.* Oil and natural gas production processes also contribute contaminants to the air, creating another potential route of exposure for humans and animals (Colborn et al. 2014; Helmig et al. 2014; Macey et al. 2014; Moore et al. 2014). Potential sources of inhalation exposure for these chemicals include evaporation from surface spills and evaporation pits, flaring at the surface, and release of chemicals during surface transfers and during processing (Colborn et al. 2014; Trimble 2012). High-level releases of chemicals are episodic (Brown et al. 2014, 2015). Elevated levels of volatile organic compounds (VOCs) such as BTEX, alkenes, alkanes, aromatic compounds, and aldehydes have been reported during drilling, production, and completion from nearby wells (Colborn et al. 2014; McKenzie et al. 2012; Roy et al. 2014; Steinzor et al. 2013), in some cases exceeding levels observed in heavily polluted inner cities (Helmig et al. 2014).

## Endocrine-Disrupting Chemicals and Oil and Gas Operations

*EDC activity of chemicals used in oil and natural gas operations.* Our laboratory has tested the estrogen and androgen receptor activities of 12 chemicals commonly used in oil and gas operations using a luminescence-based reporter gene bioassay in human cancer cells. We measured stimulation of receptors (agonist) or inhibition of positive control–induced expression (antagonist). We found 1 estrogen receptor agonist, 11 estrogen receptor antagonists, and 10 androgen receptor antagonists; several chemicals exhibited multiple receptor activities (Kassotis et al. 2014).

A 2011 analysis reported approximately 120 known or suspected EDCs out of 353 oil and gas operation chemicals with Chemical Abstract Service (CAS) numbers (Colborn et al. 2011). Importantly, only half of the known oil and gas operation chemicals had CAS numbers at that time, greatly limiting the health assessment for other chemicals used in these processes (Waxman et al. 2011). Still other chemicals remain proprietary information (Shonkoff et al. 2014; Wiseman 2011). For example, a recent study found that 67%, 37%, and 18% of assessed wells were fractured with ≥ 1, 5, or 10 proprietary chemicals, respectively (Souther et al. 2014).

*EDC activity in water near oil and natural gas operations.* We assessed the estrogen and androgen receptor activities of water samples collected from five sites in a drilling-dense region of Garfield County, Colorado, that had experienced industry-related spills or preventable discharges relative to surface and groundwater collected immediately outside of the drilling-dense region (Kassotis et al. 2014). Analysis of these samples revealed that surface and groundwater from Garfield County spill sites contained significantly elevated estrogen agonist, estrogen antagonist, and androgen antagonist activities relative to those at reference sites (Kassotis et al. 2014). Independent analytical water testing at these sites identified chemicals that we or others have shown to exhibit these same agonist and antagonist activities (discussed by Kassotis et al. 2014). Other researchers have reported estrogen agonist and androgen antagonist activities associated with oil sands and oil production wastewater (He et al. 2011; Thomas et al. 2004, 2009; Tollefsen et al. 2007).

*Concentration of oil and natural gas operation chemicals in water.* Hydraulic fracturing wastewater is reported to contain hundreds of organic chemicals (polyethylene glycols, ethoxylated surfactants, BTEX compounds, biocides, polycyclic aromatic hydrocarbons, aromatic amines, and more), with total dissolved organic carbon as high as 5.5 g/L, and many individual compounds present at > 500 mg/L and up to grams per liter concentrations (Kahrilas et al. 2015; Maguire-Boyle and Barron 2014; Orem et al. 2014; Thurman et al. 2014). A recent report analyzed publicly available data on FracFocus, an industry disclosure website (http://www.fracfocus.org/), and reported benzene ≤ 4.1% and naphthalene and ethylbenzene ≤ 0.45% of total fracturing fluid volume, resulting in milligrams per liter concentrations for these and other chemicals (Schaeffer and Bernhardt 2014).

Surface spills have been reported to contaminate groundwater with chemicals from oil and gas operations (Gross et al. 2013). Groundwater at surface spill sites contained 1.4, 2.2, 0.2, and 2.6 mg/L benzene, toluene, ethylbenzene, and xylene, respectively, and these concentrations decreased over time and distance from the spill sites (Gross et al. 2013). Sampling of groundwater in Pavillion, Wyoming, by the U.S. EPA in a region where no specific accident or spill had occurred revealed concentrations of BTEX, naphthalene, ethylene glycols, and other oil and gas chemicals at concentrations ranging from 0.01 to 8 mg/L (DiGiulio et al. 2011). Because some of these chemicals have been shown to disrupt multiple hormone receptors *in vitro* at concentrations in the micrograms per liter range (Kassotis et al. 2014), these groundwater samples contained concentrations of these chemicals within the bioactive range in our reporter gene assays. To date, few comprehensive analyses have been performed of oil and gas operation–derived chemicals in drinking-water samples.

## Potential Endocrine-Related Health Effects of Oil and Gas Operation Chemicals

*Oil and gas operation chemicals and health effects.* Evidence of potential harm from exposure to hazardous chemicals, pollutants, and emissions used in oil and natural gas operations has been reported. These reports have most often been case series involving natural experiments using quasi-experimental design and have investigated domestic animals and wildlife (Bamberger and Oswald 2012). Researchers have also begun to document in both reports and white papers the content and quantities of hazardous chemicals, pollutants, and emissions associated with these operations (Eastern Research Group and Sage Environmental Consulting 2011; Ethridge 2010; Steinzor et al. 2013; Witter et al. 2008). Concurrent with these environmental testing projects, surveys of local residents were also performed, and the reports suggested that living in close proximity to oil and gas operations has the potential to affect human and environmental health (Ferrar et al. 2013a; Rabinowitz et al. 2015; Steinzor et al. 2013; Subra 2009, 2010). At the present time, a limited number of epidemiology studies have been conducted to explore the relationship between health effects and exposure to oil and gas operation chemicals as described herein and as reviewed by Webb et al. (2014) and Werner et al. (2015).

The biological plausibility of health effects associated with exposure to hazardous chemicals, pollutants, and emissions used in oil and natural gas operations has also been explored. Many of these chemicals have documented adverse health effects in humans, are designated priority pollutants by the U.S. EPA, and/or are known or suspected EDCs (Colborn et al. 2011; Waxman et al. 2011). For example, exposure to naphthalene, a constituent of crude oil and a chemical used by industry for hydraulic fracturing processes (Waxman et al. 2011) and that has been reported in air and water near operations (Colborn et al. 2014; DiGiulio et al. 2011; Wolf Eagle Environmental 2009), can result in altered steroid hormone levels, increased reproductive abnormalities, and impaired sexual maturation in animal models and *in vitro* (Hansen et al. 2008; Pollino et al. 2009; Thomas and Budiantara 1995; Tintos et al. 2006), albeit generally at greater concentrations than those reported near these sites.

*Occupational exposures.* As with all environmental exposures, those who work around or with hazardous chemicals face significantly higher exposure risk than does the general population. The National Institute of Occupational Health and Safety (NIOSH) has published two studies for the oil and natural gas extraction industry: one about work crew exposures to respirable crystalline silica, and the other about work crew exposures to VOCs (Esswein et al. 2013, 2014). In both cases, these pilot data indicated that some workers’ exposures exceeded NIOSH and/or ACGIH safe levels (reported therein) for crystalline silica, flammable hydrocarbon emissions, and benzene.

*Reproductive effects.* Exposure to VOCs including but not limited to benzene, toluene, ethylbenzene, xylenes, and formaldehyde, all chemicals used in and produced by oil and natural gas operations (Colborn et al. 2011; Waxman et al. 2011), is associated with reproductive health effects in both humans and animals. These effects include impaired fertility and fecundity via reduced semen quality and impaired menstrual cycles as well as increased risk of miscarriage, stillbirth, preterm birth, and birth defects, as reviewed by Webb et al. (2014). A list of other adverse endocrine health effects due to exposure to single chemicals used in and produced by oil and gas operations has been assembled and is available online (http://endocrinedisruption.org/chemicals-in-natural-gas-operations/chemicals).

*Adverse pregnancy outcomes.* McKenzie et al. (2014) used spatial analysis to evaluate the likelihood of adverse pregnancy outcomes in a cohort of 12,842 live births for mothers living within 10 miles of drilling well operations compared with mothers with no drilling wells within 10 miles. Significantly increased risks for congenital heart defects [adjusted odds ratio (AOR) = 1.3; 95% confidence interval (CI), 1.2, 1.5] and neural tube defects (AOR = 2.0; 95% CI: 1.0, 3.9) were observed, but no association with oral clefts (AOR = 0.82; 95% CI: 0.55, 1.2) was observed. In contrast, a study on low birth weight that used a similar design showed mixed results (McKenzie et al. 2014; Stacy et al. 2015). In two case–control studies, maternal or paternal occupational exposure to glycol ethers (hormonally active chemicals used in fracturing fluids; Kassotis et al. 2014; U.S. EPA 2015; Waxman et al. 2011) and other chemicals (pesticides, polychlorinated compounds, phthalates, bisphenol A, alkylphenolic compounds, heavy metals, and miscellaneous agents) during pregnancy was associated with congenital malformations (Cordier et al. 1997).

*Cancer.* In a health impact assessment, McKenzie et al. (2012) used spatial modeling based on residence proximity (≤ 0.5 miles vs. > 0.5 miles) to oil and gas operations in Colorado and found an elevated cumulative cancer risk for people living near drilling wells (10 per 1,000,000 vs. 6 per 1,000,000). Two studies calculated standardized incidence ratios. One study was a cancer cluster analysis that compared the rates for several cancers in a drilling-dense Texas town with state rates using 3 years of cancer incidence data. Mokry et al. (2010) reported a statistically significantly elevated rate for breast cancer [(standardized incidence ratio (SIR) = 1.3; 95% CI: 1.1, 1.5]. The other study compared Pennsylvania counties before and after launching drilling operations. Fryzek et al. (2013) found a slightly increased rate of one cancer, central nervous system tumors (SIR = 1.13; 95% CI: 1.02, 1.25), after unconventional drilling operations began in northeast Pennsylvania (Fryzek et al. 2013).

*Limitations and data gaps.* Limitations of the above-mentioned studies are the lack of both direct exposure assessment and information on residential mobility of study participants. To date, no longitudinal study has enrolled a cohort of residents in a community that has an active oil and natural gas extraction industry so that biomarkers can be obtained in a timely manner. Known and suspected risk factors need to be collected to fully model the exposure risk. The critical route/timing of exposure for hazardous chemicals associated with oil and natural gas operations has yet to be established. Drilling wells release different amounts of air pollutants at different stages of the development and production processes (Brown et al. 2014; Colborn et al. 2014; Helmig et al. 2014; McKenzie et al. 2012), and residents, including pregnant women, may be exposed to these pollutants throughout extraction or only during specific stages. Drinking-water exposure may show considerable heterogeneity owing to the hydrogeology of undergroundwater flow associated with released natural and man-made chemicals, and limited data are available on contamination of drinking water in areas that have oil and natural gas operations.

## Recommendations

The endocrine system is designed to respond to extremely low concentrations of hormones, making it uniquely equipped to assess exposure to low levels of exogenous hormonally active contaminants. Although toxicological studies often assess adverse outcomes from high-exposure scenarios relevant to occupational exposure, endocrinological studies can assess outcomes from low-level exposure that may be more relevant to humans living near oil and natural gas operations. By combining existing *in vivo* EDC studies with knowledge of the hormone receptor activity profile of chemicals used in oil and natural gas operations, we can identify adverse health outcomes in areas where humans and animals are exposed to these chemicals for epidemiological assessment. We can then use a modified Bradford-Hill approach to assess causality between environmental exposures and adverse health outcomes, as suggested by Zoeller et al. (2014). The risks related to potential exposure and adverse outcomes in humans and wildlife populations have not been afforded complete evaluations in part because of exemptions from parts of six key federal regulatory acts that traditionally act to safeguard U.S. water sources, including the Safe Drinking Water Act and the Clean Water Act (Clean Water Act 1972; Deutch et al. 2011; Safe Drinking Water Act 1974).

Based on the hypothesis that exposure to oil and natural gas chemicals contributes to negative health outcomes, we offer the following recommendations to evaluate the risks posed to humans and wildlife: *a*) integrate endocrine-centric end points into human health assessments in areas of unconventional drilling operations; *b*) perform biomonitoring studies for chemicals and their metabolites in humans; *c*) develop an effect-directed screening approach to assess endocrine-related effects of mixtures; *d*) perform controlled laboratory animal studies of exposure to complex mixtures of oil and natural gas chemicals to assess adverse health outcomes; and *e*) perform *in vitro* bioassays to assess receptor interactions with complex mixtures.

Endocrine health assessments. We suggest incorporating an endocrine-centric component into overall human and environmental health assessments. An endocrine-centric health component would assume additivity of chemicals, an assumption that has been shown to be reasonable for chemicals acting through similar mechanisms of action (Payne et al. 2000; Rajapakse et al. 2002; Silva et al. 2002). This approach would assess common adverse endocrine end points that have been shown to result from disruption of specific hormone receptors alone and in combination, including *a*) reproductive effects (infertility, subfertility, reduced sperm counts, miscarriage, preterm birth, birth weight, puberty), *b*) developmental irregularities (cryptorchidism, hypospadias, neural tube defects, congenital heart defects), and *c*) cancer, particularly hormone-responsive types such as testicular, breast, prostate, and brain cancers (reviewed by Bergman et al. 2013; Diamanti-Kandarakis et al. 2009; Vandenberg et al. 2012; Zoeller et al. 2012).

Measurement of chemicals in humans and wildlife (biomonitoring). One of the major limitations in human risk assessment of oil and natural gas operations is the paucity of chemical exposure information, considering the number of chemicals used and the proprietary disclosure rules. Until now, most research has focused on airborne emissions (reviewed by Moore et al. 2014) and water contamination (reviewed by Rozell and Reaven 2012; Vengosh et al. 2014). Although epidemiological studies have begun to assess adverse health outcomes near drilling operations (McKenzie et al. 2014), to our knowledge, no researchers have yet published data on concentrations of oil and gas operation chemicals in humans or wildlife.

Chemical characterization is required to determine appropriate biomonitoring candidates. Recent work has detailed analytical approaches for characterizing the various classes of compounds present in hydraulic fracturing wastewater (Ferrer and Thurman 2015). We suggest that oil and gas wastewater be used to determine the presence of chemicals that can result in the observed agonist and/or antagonist responses. Initial identification should occur via reverse matching to known compound lists such as the National Institute of Standards and Technology (NIST) Spectral Search Program for the NIST/U.S. EPA/National Institutes of Health (NIH) Mass Spectral Library. These compounds can be further reverse-matched to known oil and gas operation chemicals (Colborn et al. 2011, 2014; U.S. EPA 2015; Waxman et al. 2011). Because this step may miss proprietary compounds not currently reported by industry, it should be used as a supplement to reverse-matching databases. These compounds can then be confirmed by comparing them with authentic standards. These chemicals can be further tested in bioassays to determine receptor activities and their likely presence and contribution to activities in water. These data can then guide the development of analytical methods for target compounds and their metabolites serving as biomonitoring candidates in humans living near extraction operations.

Using effects-directed analysis to identify chemicals responsible for EDC activity. Analytical identification of hormonally active chemicals present in both water and air must be performed to better characterize source and exposure and to assess risk. Whenever possible, analysis of complex environmental samples should be performed using an effects-directed analysis approach (Burgess et al. 2013; Liscio et al. 2014; Rostkowski et al. 2011) coupled with a response–balance approach (Cargouët et al. 2004; Schriks et al. 2010; Sun et al. 2008).

This effects-directed/response–balance approach should target the most hormonally active samples from drilling regions (as well as from reference sites to eliminate background activity/chemicals) for chemical fractionation and testing. These procedures should include orthogonal separations and screening of the resulting fractions in bioassays to refine and isolate bioactive chemicals. Refined fractions can then be analyzed using the mass spectrometry (MS) tools described below and recently reported (Ferrer and Thurman 2015) to help identify chemicals responsible for observed activities. Once candidate chemicals have been identified, authentic standards may be used to confirm the MS identification and the bioactivity observed in bioassays. This method has been used successfully to identify novel bioactive compounds and represents the best approach for characterizing the EDCs that are most responsible for observed activities (Liscio et al. 2014; Rostkowski et al. 2011). Finally, biological activity can be coupled with chemical concentrations obtained from environmental monitoring to determine relative contributions to observed receptor activities, as has been described by others (Cargouët et al. 2004; Schriks et al. 2010; Sun et al. 2008).

EDC-centric laboratory animal health assessments. Laboratory animal models can and should be used to test for causal relationships between exposure and negative health outcomes that might be expected in drilling-dense regions. Humans and wildlife living in these regions are likely exposed to oil and gas operation chemicals during different developmental windows, and known critical periods such as prenatal, perinatal, childhood, and puberty should be targeted. Studies of adult exposure should also be performed to assess occupational exposure and chronic exposure at environmentally relevant levels encountered by nearby residents. We further recommend that the route of exposure remain as relevant as possible. Likely exposure to chemicals may occur through oral, dermal, and/or inhalation routes, and parameters such as volatility and partition coefficients will help determine which exposure routes are of the highest concern for individual chemicals. Route of exposure is crucial to understanding health effects because varying routes of exposure can result in very different bioavailability of EDCs, as has recently been described for bisphenol A (Gayrard et al. 2013; Hormann et al. 2014; vom Saal and Welshons 2014). Adverse health outcomes that should be targeted are described above in both the section entitled “Potential Endocrine-Related Health Effects of Oil and Gas Operation Chemicals” as well as in our recommendation regarding endocrine health assessments and are known to result from exposure to EDCs (reviewed by Bergman et al. 2013; Diamanti-Kandarakis et al. 2009; Vandenberg et al. 2012; Zoeller et al. 2012); many protocols have been described for the evaluation of these end points (Diamanti-Kandarakis et al. 2009; Schug et al. 2013; U.S. EPA 2009a, 2009b, 2009c; Zoeller et al. 2012). These data can provide important information for further refining human epidemiological studies as well as studies on pets and wildlife populations, which have recently been shown to be affected by endocrine health concerns (Bamberger and Oswald 2012, 2014, 2015; Grant et al. 2015; Papoulias and Velasco 2013; Slizovskiy et al. 2015).

Bioassays for complex mixtures. With approximately 1,000 chemicals used in and produced by oil and gas operations (U.S. EPA 2015), there is a critical need for methods to assess the EDC activity of these complex mixtures. Methods of assessing the activity and potential health risks of mixtures that can appropriately address the interplay between receptor systems are limited. Observed outcomes *in vivo* can often be the result of disruption of several hormone receptor systems by single chemicals or by mixtures. Statistical modeling (Orton et al. 2012), *in vitro* and *in vivo* assays (Silva et al. 2002), quantitative structure analysis (Nishihara et al. 2000), gene expression (Richter et al. 2014), and other tools have been used to assess a number of laboratory-defined mixtures that interact with single hormone receptors.

Modeling complex mixtures can greatly reduce the number of independent tests that need to be performed when assessing toxicity. For example, Bertin et al. used a neural networking model to assess mixture toxicity, achieving a predictive model with approximately 10% of actual interactions tested (Bertin et al. 2013). However, despite clear successes with relatively uncomplicated mixtures, analysis of more complicated mixtures appears to be beyond current capabilities (Kortenkamp et al. 2014; Orton et al. 2012) owing to insufficient knowledge of interreceptor interactions and indirect chemical–receptor interactions (Kortenkamp et al. 2014). An additional concern involves indirect interactions between chemicals and receptors. For example, interaction with the aryl hydrocarbon receptor can result in the activation of cytochrome P450 enzymes, which are well known to alter endogenous and exogenous chemical metabolism and therefore exposure (Anzenbacher and Anzenbacherová 2001; Markowitz et al. 2003). Inactive chemicals can be metabolized into active metabolites, resulting in mixtures of inactive chemicals that can act as agonists or antagonists in mixtures only (Gauger et al. 2007). Improved characterization of these interactions will provide a clearer understanding of the utility models can provide towards assessing *in vivo* outcomes, as well as their limitations.

Because it is not possible to test all combinations of chemicals *in vitro* and/or *in vivo*, we recommend performing guided *in vitro* and *in vivo* research that focuses on receptor interactions. We suggest that reporter gene assays be used for *in vitro* testing because of their low cost, ease of use, reliability, high sensitivity, and ease of adapting for multiple receptor systems (Naylor 1999; Rajapakse et al. 2002; Silva et al. 2002; Soto et al. 2006). Similar assays including yeast receptor screens [yeast estrogen screen (YES), yeast androgen screen (YAS), etc.] tend to be less robust and less sensitive, albeit less susceptible to toxicity, whereas cell proliferation assays (E-SCREEN, A-SCREEN, etc.) are equally sensitive and, unlike reporter gene assays, can measure nongenomic effects through cell-surface receptors; however, they are generally less applicable for diverse receptor testing (Leusch et al. 2010). Current high-throughput assay options such as Tox21 or ToxCast^TM^ are of great use as diverse first-pass screens for individual compounds, although it is unclear whether they will be helpful in the assessment of complex mixtures (Filer et al. 2014; Tice et al. 2013). Rather than the single-receptor tests used by these systems, assessing chemicals and mixtures of chemicals in controlled multiple-receptor systems is critical to understanding and accounting for receptor interplay.

Improvement of the utility of *in vitro* assay systems should take place in several steps. First, receptor interaction can be assessed through testing positive controls in both the presence and the absence of other receptors. Ideally, this testing should be done across several cell lines to identify chemical impingement on receptor interactions and tissue-specific comodulators. Once multiple-receptor experiments are carried out with single chemicals, simple mixtures with clearly defined receptor activity profiles can be introduced to determine how simultaneous interactions with several receptors can modulate responses. Further work should be coupled with *in vivo* experiments to understand these interactions in a whole-animal model and to confirm *in vitro* multiple-receptor results.

## Potential Implications

Recent analyses of the potential contributions of EDC exposures to adverse endocrine health outcomes, such as obesity, cancers (particularly hormone-dependent), reproduction/infertility, metabolic diseases, and developmental abnormalities, suggest that EDC exposures account for an estimated 1.8% to 40% of societal health care costs (Hunt and Ferguson 2014; Olsson 2014; Trasande 2014). More recently, a suite of studies estimated the potential health care costs for the European Union (EU) due to EDC exposures: neurobehavioral deficits and disorders (> 150 billion euros; Bellanger et al. 2015), obesity and diabetes (> 18 billion euros; Legler et al. 2015), and male reproductive disorders and diseases (> 15 billion euros; Hauser et al. 2015). Altogether, the median cost to the EU for EDCs with the highest probability of causation was estimated at 157 billion euros per year (Trasande et al. 2015). Whereas exposure to oil and gas operation chemicals individually would likely result in only a fraction of these costs, increasing exposure to additional hormonally active chemicals is a cause for concern given the additive nature of many of these receptor systems. As such, there are potentially large financial implications for exposure to EDCs from their use in oil and gas operations.

## Conclusions

Herein, we have provided a series of recommendations that will allow scientifically defensible, accurate assessments of the potential endocrine-related risks from chemical exposure associated with oil and natural gas operations. We present these recommendations in light of the growing body of information regarding both chemical concentrations in the environment and adverse health outcomes reported in humans and in wildlife. We suggest that these approaches will lead to improved information for resource management decisions and will ultimately protect and improve human health.
